# The miRNA Pathway Controls Rapid Changes in Activity-Dependent Synaptic Structure at the *Drosophila melanogaster* Neuromuscular Junction

**DOI:** 10.1371/journal.pone.0068385

**Published:** 2013-07-02

**Authors:** Katherine R. Nesler, Robert I. Sand, Breanna A. Symmes, Sarala J. Pradhan, Nathan G. Boin, Anna E. Laun, Scott A. Barbee

**Affiliations:** 1 Department of Biological Sciences and Eleanor Roosevelt Institute, University of Denver, Denver, Colorado, United States of America; 2 Molecular and Cellular Biophysics Program, University of Denver, Denver, Colorado, United States of America; 3 Colorado Intellectual and Developmental Disabilities Research Center (IDDRC), University of Colorado Anschutz Medical Campus, Aurora, Colorado, United States of America; University of Edinburgh, United Kingdom

## Abstract

It is widely accepted that long-term changes in synapse structure and function are mediated by rapid activity-dependent gene transcription and new protein synthesis. A growing amount of evidence suggests that the microRNA (miRNA) pathway plays an important role in coordinating these processes. Despite recent advances in this field, there remains a critical need to identify specific activity-regulated miRNAs as well as their key messenger RNA (mRNA) targets. To address these questions, we used the larval *Drosophila melanogaster* neuromuscular junction (NMJ) as a model synapse in which to identify novel miRNA-mediated mechanisms that control activity-dependent synaptic growth. First, we developed a screen to identify miRNAs differentially regulated in the larval CNS following spaced synaptic stimulation. Surprisingly, we identified five miRNAs (miRs-1, -8, -289, -314, and -958) that were significantly downregulated by activity. Neuronal misexpression of three miRNAs (miRs-8, -289, and -958) suppressed activity-dependent synaptic growth suggesting that these miRNAs control the translation of biologically relevant target mRNAs. Functional annotation cluster analysis revealed that putative targets of miRs-8 and -289 are significantly enriched in clusters involved in the control of neuronal processes including axon development, pathfinding, and growth. In support of this, miR-8 regulated the expression of a *wingless* 3′UTR (*wg* 3′ untranslated region) reporter *in vitro*. Wg is an important presynaptic regulatory protein required for activity-dependent axon terminal growth at the fly NMJ. In conclusion, our results are consistent with a model where key activity-regulated miRNAs are required to coordinate the expression of genes involved in activity-dependent synaptogenesis.

## Introduction

The establishment of long-lasting changes in synapse structure and function requires the rapid regulation of spatial and temporal gene expression in response to neural stimulation. New evidence indicates that the miRNA pathway plays an important role in the control of these processes [1]. miRNAs are abundant small regulatory RNAs that postranscriptionally repress the expression of target mRNAs, usually by binding to sequences in their 3′ UTRs. With the exception of the “seed sequence” (positions 2–8 of the miRNA), miRNAs bind to target mRNAs with only partial complementarity. This allows each individual miRNA to bind to and, potentially, coordinate or fine-tune the expression of 10s to 100s of target mRNAs [2]. In neurons, miRNAs are involved in the control of diverse cellular processes ranging from dendrite spine formation and/or function to the control of synaptic plasticity [3]. As such, dysregulation of key neuronal miRNA expression is associated with several human neurological disorders [4].

Despite the apparent importance of miRNAs in long-term synaptic plasticity, only a handful of activity-regulated miRNAs and *bona fide* mRNA targets have been identified. First, in a genome-wide screen, the murine miR-29a/b cluster was found to be significantly upregulated by exposure to neurostimulants [5]. miR-29a/b regulates dendritic spine morphology by controling actin cytoskeleton remodeling via down-regulation of Arpc3, a component of the ARP2/3 actin nucleation complex. Second, in a genome-wide screen to identify miRNA targets of the cAMP-response element binding protein (CREB), rat miR-132 was found to be up-regulated by brain derived neurotrophic factor (BDNF) and synaptic activity [6]. miR-132 controls dendritic plasticity by down-regulating the expression of p250GAP, a GTPase activating protein that regulates the Rac1/PAK1 pathway [7], [8]. Third, in a more targeted screen, the transcription factor myocyte enhancing factor 2 (Mef2) was found to be necessary and sufficient for activity-dependent up-regulation of the rat brain-specific miR-379–410 cluster [9]. One miRNA in this cluster, the dendritically localized miR-134, regulates dendrite morphogenesis by controlling the expression of the conserved translational repressor, Pumilio2 [10]. Finally, in a microarray-based screen, induction of long-term potentiation (LTP) was found to specifically up-regulate the expression of miR-188 in rat hippocampal neurons [11]. miR-188 controls dendritic spine development and synapse structure by negatively regulating expression of the semaphorin-3F receptor, neuropilin-2 (Nrp-2). Importantly, when taken together, these data support a model where specific activity-regulated miRNAs coordinate the expression of mRNAs encoding for proteins involved in the control of activity-dependent synaptic plasticity.

Do miRNAs regulate activity-dependent changes in synapse structure or function in *Drosophila*? To directly address this question, we developed a screen using the glutamatergic larval NMJ as a model synapse to identify and characterize novel miRNAs involved in the control of activity-dependent synaptic growth. Previous work at the NMJ has shown that acute stimulation with high K^+^- or Channelrhodopsin-2 (ChR2) light-induced spaced depolarization results in rapid activity-dependent changes in both synapse structure and function [12]. This new synaptic growth is above and beyond normal developmental scaling of the NMJ to its target muscle. Instead, it is thought to be analogous to the activity-dependent changes observed in dendritic spines of cultured hippocampal neurons [12], [13]. In subsequent studies, this system has begun to reveal novel mechanisms that control activity-dependent structural synaptic plasticity [14], [15].

We hypothesized that spaced synaptic stimulation (sufficient to induce lasting effects) will first control the expression of a subset of neuronal miRNAs. In turn, these miRNAs will regulate the translation of key target mRNAs involved in the presynaptic control of axon terminal growth at the NMJ. Interestingly, using a sequential miRNA microarray and real-time quantitative PCR (RT-qPCR)-based screen, we identified five mature miRNAs (miRs-1, -8, -289, -314, and -958) whose levels are significantly downregulated following acute spaced stimulation. Neuronal misexpression of three of these miRNAs (miRs-8, -289, and -958) is sufficient to prevent activity-dependent synaptic growth at the NMJ. Combined *in silico* target analysis and functional annotation analysis revealed that several predicted mRNA targets for co-regulation by activity-regulated miRNAs have functions in the control of synapse structure and/or function. As proof of concept, miRs-8, -289, and/or -958 can repress the expression of two putative target mRNAs in an *in vitro* luciferase reporter assay. These mRNAs encode for Wg and the *Drosophila* leukocyte-antigen-related-like protein, Lar [12], [16]. Consistent with these observations, knockdown of presynaptic Wg and Lar by RNAi resulted in the specific suppression of new synaptic growth in response to activity [12]. Together, these data suggest that miRs-8, -289, and -958 are involved in activity-dependent processes. However, further analysis is required to confirm relevant miRNA/mRNA interactions.

## Materials and Methods

### Drosophila stocks

All fly stocks were raised at 25°C on standard Bloomington media with the exception of ChR2-expressing fly lines. For those experiments, flies were raised on standard Bloomington food supplemented with 100 mM all-trans retinal to facilitate ChR2 activity *in vivo* [17], [18]. *Canton S, w^1118^, C380-Gal4, UAS-ChR2, UAS-wg^HMS00794^*, and *UAS-lar^HMS00822^* fly strains were all obtained from the Bloomington *Drosophila* Stock Center. *miR-8^Δ1^* and *miR-8^Δ2^* deletion flies were a gift from S. Cohen [19]. *UAS-miR-8* *SP #10* (miR-8 sponge) flies were a gift from D. Van Vactor [20].

### Activity Assays and Immunohistochemistry

ChR2 stimulation was executed using a homemade apparatus that was built based on general descriptions [12], [18]. Briefly, non-wandering third instar larvae were collected, washed twice with HL-3 haemolymph-like dissection buffer [21], and placed into the stimulation chamber. The larvae received five 5 min stimulation steps with each step composed of a series of cycles (2 sec on and 3 sec off) where emission of 470 nm light from two blue LEDs (Luxeon V; Luxeon Star) was controlled by a pulse stimulator (A-M Systems) essentially as previously described in [12]. Each stimulation step was separated by a 15 min rest period. A 134 min resting step followed the final stimulation. Control larvae were exposed to identical conditions but not subjected to stimulation cycles. Upon completion of the ChR2 stimulation paradigm, larval preparations were either processed for NMJ analysis or the CNS was explanted for RNA isolation.

The high K^+^ stimulation paradigm was performed essentially as previously described [12], [14], [15]. Briefly, wandering third instar larvae were collected and semi-dissected in HL-3 leaving the CNS intact. The larvae were then subjected to a stimulation paradigm where they were treated with high K^+^ (90 mM KCl) HL-3 adjusted for osmolarity [22] in a pattern of 2, 2, 2, 4 and 6 min pulses (5x high K^+^ stimulation). Alternatively, larvae were treated with high K^+^ HL-3 in a pattern of 2, 2, and 2 min pulses (3x high K^+^ stimulation). Each stimulation step was separated by a 15 min rest period in normal HL-3 (5 mM KCl) and a 74 min resting step followed the final stimulation. Pseudostimulated control larvae were subjected to an identical protocol except the high K^+^ HL-3 was replaced with normal HL-3 during each pulse step. Upon completion of the high K^+^ stimulation paradigm, larval preparations were either processed for NMJ analysis or the CNS explanted for RNA isolation.

### Microarray Analysis

For miRNA array analysis, two genotypes (*w^1118^* and *UAS-ChR2 x C380-Gal4*) were used. ChR2-expressing larvae were taken through either the light- or mock-stimulation paradigm. At the end of each paradigm, 30–50 larval CNS (ventral ganglia+optic lobes) samples were manually dissected, eye imaginal discs and residual body wall removed, and collected in lysis buffer on ice. Total RNA was extracted using the miRCURY RNA Isolation kit (Exiqon) yielding ∼125 ng of total RNA per CNS. RNA was eluted with 40 µl of RNAse free water (Ambion), flash frozen, and stored at −80°C. miRNA microarray profiling was carried out at Exiqon using standard protocols. Briefly, each RNA sample was labeled with Hy3 and a common reference standard with Hy5 using the miRCURY LNA Array power labeling kit (Exiqon). The common reference sample consisted of equal amounts of RNA from *w^1118^,* ChR2 light, and ChR2 mock-stimulated CNS samples. The Hy3-labeled samples and the Hy5-labeled reference RNA sample were mixed pair-wise and hybridized to the miRCURY LNA array version 11.0 Other Species (Exiqon), which contains capture probes targeting all *Drosophila* miRNAs registered in miRBASE version 14.0. Exiqon analyzed all miRNA array data. Briefly, image analysis was carried out using the ImaGene 8.0 software (BioDiscovery, Inc.). The quantified signals were background corrected (Normexp with offset value 10) and normalized using the global Lowess (locally weighted scatterplot smoothing) regression algorithm [23]. Relative expression levels were calculated as the log_2_ normalized signal intensity between the Hy3 and Hy5. Exiqon determined the presence or absence of specific miRNAs in each sample. Changes in expression levels (fold changes) were calculated between the *w^1118^*, ChR2 light-, and ChR2 mock-stimulated groups.

### RT-qPCR Analysis

For RT-qPCR array analysis, three biological replicates of one genotype (*Canton S*) and two treatment groups (high K^+^- or mock-stimulation) were used. At the end of each paradigm, 40 larval CNS (ventral ganglia+optic lobes) samples were manually dissected, eye imaginal discs and residual body wall removed, and collected in lysis buffer on ice. Total RNA was extracted using the miRNeasy RNA Isolation kit (Qiagen) yielding ∼150 ng of total RNA per CNS. RNA was eluted with 40 µl of RNAse free water (Ambion), flash frozen, and stored at −80°C. Prior to freezing, a small aliquot was removed for quality control. RNA quality was assessed using an Experion automated electrophoresis system (BioRad) and samples with a RNA quality indicator (RQI) score <7 discarded and re-extracted. ∼6 µg of total RNA from each replicate was simultaneously converted to cDNA using the miScript reverse transcriptase kit (Qiagen). Three technical replicates of each biological replicate were then amplified using miScript SYBR Green PCR Kit (Qiagen). Primer assays were obtained for the 79 mature *Drosophila* identified in the larval CNS by miRNA array (Qiagen). Melt curve analysis indicated that 14 of these primers amplified non-specific PCR product and, therefore, were not included in subsequent analysis. Three primer assays failed to amplify product in all biological replicates and were therefore excluded. The results of this analysis were normalized to the U1 snRNA (verified by BestKeeper software v1.0) [24]. All RT-qPCR assays were performed using an iCycler thermocycler equipped with the iQ5 real-time PCR detection system hood and controlled by the iQ5 optical system software v1.2 (Bio-Rad). Results from three technical replicate were averaged to generate C_t_ values for each biological replicate. Then, analysis of differential fold change based on C_t_ results was performed using the Livak (ΔΔC_t_) method [25]. Changes in relative miRNA expression levels were calculated between the high K^+^ and mock stimulated groups.

Both the microarray and RT-qPCR data can be found in the Gene Expression Omnibus (GEO) of NCBI through accession number GSE43945.

### Construction of Transgenic Lines

To generate pri-miRNA expressing transgenes, PCR primers were designed to amplify a sequence ∼200 nt upstream and downstream of each miRNA hairpin from genomic DNA as described [26]. PCR products were cloned into pENTR and then into the 3′UTR of mCherry in pUASM using the Gateway cloning system (Invitrogen). All constructs were sent to Bestgene, Inc. to make and balance transgenic fly lines.

### NMJ Morphological Analysis

To study NMJ morphology, third instar larvae were dissected in calcium-free HL-3 and body wall preparations were processed as previously described [15], [21]. Primary antibodies used were mouse anti-Dlg (Developmental Studies Hybridoma Bank) and goat anti-HRP-Dylight 594 (Jackson Labs). The secondary antibody used was Alexa 488-conjugated anti-mouse IgG (Molecular Probes). Larvae were imaged at muscle 6/7 in abdominal segment 3 on an Olympus FluoView FV1000 scanning confocal microscope. All images were obtained using either a 60X (N.A. 1.35) or 100X objective (N.A. 1.4) and generated from stacks collected at intervals of 0.8 µM. These images were combined using FV1000 imaging software. Analysis of boutons and ghost boutons was done essentially as described [12], [27]. Unless otherwise indicated (see individual figures and/or figure legends), for each genotype and/or treatment group, a minimum of 20 NMJs was imaged. Unless otherwise indicated, these represent paired NMJs from both hemisegments of ≥10 larvae. All images were randomized and scored blindly using the Cell Counting plugin for ImageJ v1.45 (NIH). There were no obvious differences in muscle size between either genotypes or treatment groups.

### miRNA Target Analysis

Putative mRNA targets of miRNAs were identified using the miRecords online target prediction resource, restricting lists to targets predicted by three or more databases (http://mirecords.biolead.org) [28]. For functional annotation cluster analysis, we uploaded each list of putative target genes into the DAVID bioinformatics resource (http://david.abcc.ncifcrf.gov; Database for Annotation, Visualization and Integrated Discovery) [29], [30]. All gene names were converted to a Flybase gene ID using the “Gene ID Coversion Tool”. We used default parameters (medium stringency) and enrichment scores. Benjamini-Hochberg corrected p-values were calculated by DAVID software. Non-neuronal-related clusters were excluded from further analysis.

### Cell Culture, Transfections, and Luciferase Assays

Plasmids for luciferase reporter assay experiments are identical to those described in [31] except that the firefly luciferase (Fluc) 3′UTR reporter and miRNA overexpression plasmids have been converted into Gateway destination vectors (Invitrogen). All cloned 3′UTRs contained their endogenous poly(A) signals. All transfections were performed in three biological replicates in 6-well plates using Effectene transfection reagents (Qiagen). The transfection mixtures contained 0.1µg of the Fluc 3′UTR reporter plasmid, 0.4µg of a *Renilla* luciferase (Rluc) transfection control, and 0.5µg of either a miRNA expression vector or empty vector control. Cells were incubated for three days at room temperature and then luciferase activity for each biological replicate measured in three technical replicates on a Synergy HT microplate reader (Biotech) using a dual-luciferase reporter assay system (Promega).

### Statistical Analysis

All statistical analysis (the specific tests are indicated in the corresponding figure legends) including graphing was performed using Prism v6.0 (GraphPad software) and statistical significance was determined to be at p<0.05. Where indicated, data are normalized to controls and are presented as mean ± SEM. In the NMJ experiments, the numbers indicated in the columns of each graph are the number of individual NMJs from which measurements were taken for that genotype or treatment group. For activity assay experiments, statistical outliers were removed from analysis using the ROUT method (robust regression and outlier removal; Q = 1%) [32].

## Results

### Acute Spaced Stimulation Induces Rapid Synaptic Growth

**Figure 1:**
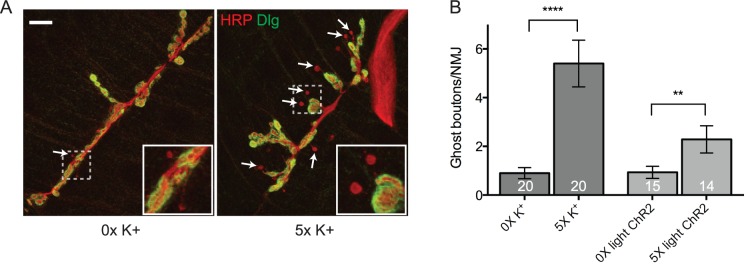
Acute spaced stimulation induces the formation of undifferentiated ghost boutons. (A) A representative *Canton S* third-instar NMJ at larval muscle 6/7 in abdominal segment 3 that was double stained with antibodies against the presynaptic membrane marker, horseradish peroxidase (HRP; red) and postsynaptic discs large (DLG; green) after being subjected to 0x (control) or 5x high K^+^ (90 mM) spaced depolarization. Arrows point to ghost boutons (HRP+ DLG-). Each inset corresponds to the indicated region (dashed box) and is blown up to help visualize ghost boutons (red HRP+ presynaptic extensions). Scale bar = 20 µM. (B) Quantification of the number of ghost boutons per NMJ in *Canton S* (high K^+^ stimulated) or animals expressing the transgenic light-gated ion channel, ChR2, in motor neurons (light stimulated; the *UAS-ChR2* line was crossed to *C380-Gal4*). These data are highly consistent with published results [12], [14], [15]. 1–2 unpaired NMJs per larval treatment group. Error bars indicate the mean ± SEM. STATISTICS: Student’s t-test. ** p<0.01 and **** p<0.0001.

In order to identify miRNAs that are differentially expressed following synaptic activity, we took advantage of two spaced training paradigms that have previously been shown to induce activity-dependent synaptic growth at the larval NMJ. First, spaced high (90 mM) K^+^ stimulation leads to the robust induction of presynaptic structures called “ghost boutons” [12]. Ghost boutons are extensions of axon terminals that contain synaptic vesicles but lack active zones and postsynaptic structures [33]. In our hands, we see ∼1 ghost bouton per NMJ in “mock” stimulated *Canton S* larvae (Figure 1A–B). Following spaced high K^+^ depolarization, we see 4–6 ghost boutons per NMJ representing an approximately 6-fold increase (Figure 1A–B; p<0.0001). Second, to account for potential non-physiological phenotypes that might result from global high K^+^ stimulation, we adapted an optogenetic approach to specifically stimulate synaptic activity in larval motor neurons [12], [17], [18]. In these experiments, the inducible transgenic light-activated ion channel, Channelrhodopsin-2 (ChR2), was driven using a motor neuron-specific Gal4 driver (*UAS-ChR2 x C380-Gal4*). As with high K^+^ stimulation, unstimulated control animals contain ∼1 ghost bouton per NMJ (Figure 1B). In contrast, this number increases to only ∼2 ghost boutons following spaced light stimulation representing an approximately 2.5-fold increase (Figure 1B; p<0.01). While light-induced spaced stimulation of ChR2-expressing larvae was clearly less robust, it was statistically significant and consistent with published results [12].

### Identification of miRNAs Expressed in the Larval Central Nervous System

**Figure 2:**
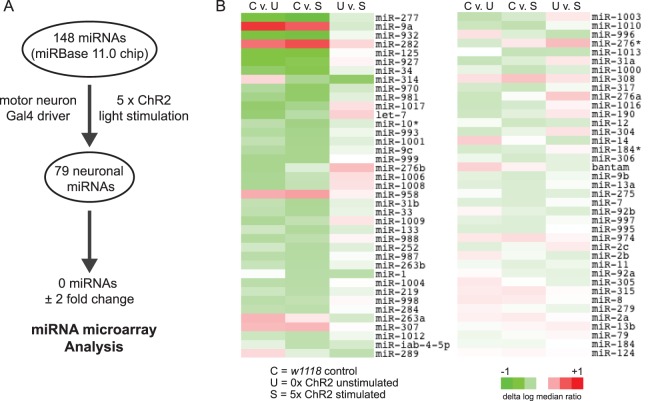
miRNA expression profile of the *Drosophila* larval CNS. (A) Flowchart showing miRNA microarray analysis. The expression of 79 mature miRNAs was detected in the *Drosophila* larval CNS. (B) Heat map showing fold-change of miRNAs in the CNS of *w^1118^* (control or “C”), ChR2 mock stimulated (unstimulated or “U”), and ChR2 light stimulated (stimulated or “S”) larvae. No neuronal miRNA exhibited a greater than 2-fold change in expression levels following ChR2 light stimulation. The largest differences observed were between the *w^1118^* and *C380-Gal4 x UAS-ChR2* genotypes.

We next sought to identify neuronal miRNAs that were differentially expressed in response to acute spaced synaptic stimulation. In initial experiments, we used light-induced spaced stimulation of ChR2-expressing larvae as our activity paradigm (Figure 1B). RNA was isolated from the dissected CNS of control (*w^1118^*) and unstimulated (0x mock) or stimulated (5x light) third instar larvae. Using a microarray containing 148 *Drosophila* miRNAs, we found that 79 were expressed in the larval CNS (Figure 2A–B; the microarray covered 35% of the 426 *Drosophila melanogaster* mature miRNAs in miRBase 19.0). Surprisingly, none of these mature miRNAs exhibited a 2-fold change in expression following acute spaced stimulation (Figure 2A–B; “U v. S” column; the 2-fold threshold is our arbitrary cutoff for biological significance). The failure to identify activity-regulated miRNAs in this assay may be due to a combination of circumstances. First, while arguably more physiological, the light-induced spaced stimulation of ChR2-expressing larvae resulted in only a modest increase in ghost bouton formation (Figure 1B). Second, by driving expression of ChR2 in only a subset of cells in the CNS (i.e. motor neurons), it may be difficult to identify significant changes in miRNA levels given background noise from unstimulated neurons. Interestingly, the greatest differences we observed were between the control for genetic background (*w^1118^*) and the ChR2-expressing line. We identified eight miRNAs that exhibited between a 1.5- and 1.9-fold change in relative expression levels (Figure 2B; “C vs. U” and “C vs. S” columns; Table S1). This result correlates with a significant increase in the total number of synaptic boutons observed at inbred *w^1118^* NMJs when compared to more wild type genotypes (our unpublished observation). Perhaps these eight miRNAs are, at least in part, involved in the control of synaptic growth during larval development.

### Identification of Activity-regulated miRNAs

**Figure 3:**
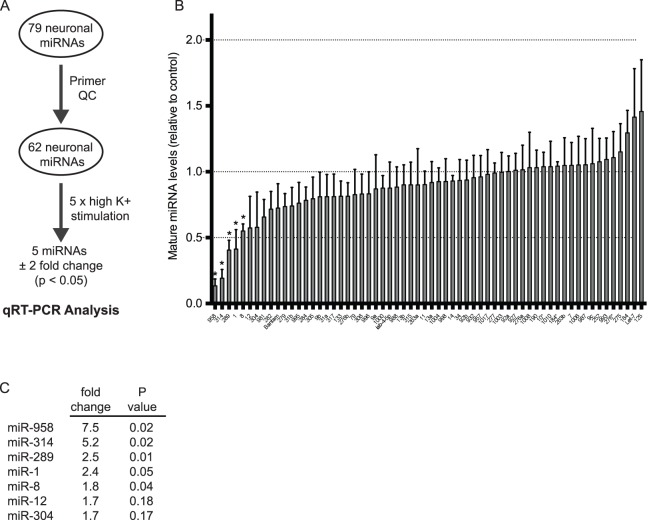
miRs-1, -8, -289, -314, and -958 are rapidly downregulated by activity. (A) Flowchart showing RT-qPCR analysis. 62 of 79 mature neuronal miRNAs were screened by RT-qPCR (see methods). (B) Five miRNAs were determined to exhibit either a 2-fold or statistically significant downregulation in expression levels following spaced high K^+^ depolarization. Error bars indicate the mean ± SEM. STATISTICS: One-way ANOVA with a Tukey’s post-hoc test (n = 3 replicates). * p<0.05. (C) Table showing fold downregulation and p-values for each activity-regulated miRNA. While not statistically significant, miRs-12 and -304 show a 1.7-fold activity-dependent downregulation. miRs-12 and -304 are both located in the miR-12/304/283 cluster.

While we did not find any differentially expressed miRNAs using optogenetics, we did identify the subset of miRNAs that were expressed in the larval CNS (Figure 2B). This allowed us to modify our approach in two important ways. First, we switched to the significantly more robust spaced high K^+^ depolarization paradigm to globally stimulate activity in the larval CNS (Figure 1A–B). Second, we developed a RT-qPCR assay to identify changes in mature miRNA levels following high K^+^ stimulation. After primer pair quality control, we were able to screen 62 of 79 neuronal miRNAs using this approach (Figure 3A; Table S2). RNA was isolated from the dissected CNS of wild-type *Canton S* unstimulated (0x mock) or stimulated (5x high K^+^) third instar larvae and comparatively analyzed by RT-qPCR. From this screen, we identified five miRNAs that exhibited either a 2-fold or statistically significant change in mature miRNA levels compared to mock stimulated controls (Figure 3A–C; p<0.05). Interestingly, we found that all five activity-regulated miRNAs (miRs-1, -8, -289, -314, and -958) were rapidly downregulated by spaced high K^+^ depolarization (within ∼70 min from the last stimulation; Figure 3B–C). The next two miRNAs that showed the strongest activity-dependent decrease belong to the miR-12/304/283 cluster (Figure 3C; miRs-12 and -304 are both downregulated 1.7-fold; p>0.05). Despite being part of this cluster, miR-283 was not detected in the larval CNS (Figure 2B; Table S1). This is consistent with published evidence that, while expression of miRs-12 and -304 strongly correlate to one another, the expression profile of miR-283 does not correlate to either miRs-12 or -304 [34]. Although our miR-12 and miR-304 results were not statistically significant, the observation that two miRNAs potentially under common transcriptional control are equally affected by activity provides strong support for our observations. In further support, while spaced high K^+^ depolarization was substantially more robust, the results from the RT-qPCR assay correlated significantly with those from light-induced ChR2 miRNA microarray analysis (Figure S1; r = 0.37; p<0.001).

### miR-8, miR-289, and miR-958 Negatively Regulate Activity-dependent Growth

**Figure 4:**
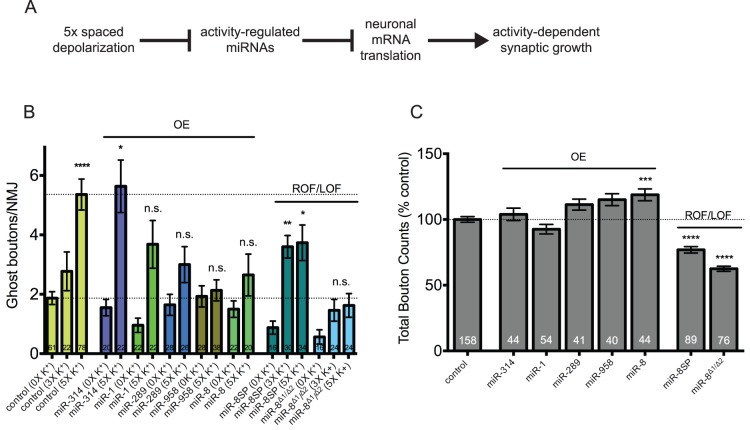
miRs-8, -289, and -958 are required for activity-dependent ghost bouton formation at the larval NMJ. (A) Our working model for miRNA-mediated control of activity-dependent synaptic growth at the larval NMJ. In an unstimulated motor neuron, key activity-regulated miRNAs negatively regulate the expression of target mRNAs involved in the control of activity-dependent synaptic growth. In contrast, our data suggests that acute spaced stimulation results in the rapid downregulation of mature miRNA levels. This would result in the increased translation of target mRNAs and subsequent rapid activity-dependent synaptic growth. (B) Transgenic pri-miRNAs for miRs-1, -8, -289, -314, or -958 were misexpressed in larval motor neurons using the *C380-Gal4* driver (e.g. genotype *C380-Gal4/+; UAS-pri-miR/+*). Also examined were miR-8 knockdown (*C380-Gal4/+; UAS-miR-8SP/+*) or loss-of-function (*mir-8^Δ1^/^Δ2^*) lines. Ghost boutons per NMJ were quantified in indicated genotypes under conditions of mock (0x), intermediate (3x) or high (5x) K^+^ stimulation as indicated. Compared to their matched 0x unstimulated controls, the misexpression of miRs-8, -289, and -958 did not result in a significant increase in ghost bouton numbers following 5x high K^+^ stimulation. In contrast, miR-8 knockdown (*UAS-miR-8SP*) and deletion (*mir-8^Δ1^/^Δ2^*) larvae showed an enhanced ability to respond to intermediate stimulation. (C) Quantification of the combined number of synaptic boutons (stimulated and unstimulated) at the same NMJs assayed in (B). No significant difference in total bouton number was observed between unstimulated and stimulated NMJ within any miRNA genotype (data not shown). Note that only miR-8 has a significant effect on total bouton numbers when compared to controls (*C380-Gal4/+*). This suggests that miR-8 has a function in both ghost bouton maturation and development. (B-C) OE = overexpression; ROF = reduction-in-function; LOF = loss-of-function. The numbers located at the bottom of each column indicate the number of NMJs analyzed for each genotype and/or treatment group. Except in *C380-Gal4/+* there are 2 paired NMJs per treatment group. Error bars indicate the mean ± SEM. (B-C) STATISTICS: Kruskal-Wallis multiple comparison analysis with a Dunn’s post-hoc test. n.s. = not significant * p<0.05 ** p<0.01 *** p<0.001 **** p<0.0001.

The rapid downregulation of mature miRNA levels following acute spaced depolarization is correlative and only suggests that activity-regulated miRNAs are involved in the control of activity-dependent synaptic growth. However, this observation did allow us to propose the following simple model (Figure 4A). In response to acute spaced synaptic stimulation (high K^+^ or light-stimulated ChR2), levels of key activity-regulated miRNAs are reduced. In turn, this results in the increased translation of target mRNAs encoding for proteins involved in the control of activity-dependent growth at the NMJ. Based on this model, we postulated that, if miRs-1, -8, -289, -314, and/or -958 were involved in this process, then misexpression of transgenic miRNAs that could not be transcriptionally downregulated by activity would prevent ghost bouton formation. To test this hypothesis, we drove expression of a primary miRNA (pri-miRNA) construct for each activity-regulated miRNA in larval motor neurons. Tissue-specific misexpression of transgenic pri-miRNAs like this has been shown to be capable of inducing very specific and functionally relevant mutant phenotypes [35]. Transgenic larvae were subjected to either mock stimulation or spaced high K^+^ depolarization. In pseudostimulated controls, we observed ∼2 ghost boutons per NMJ (Figure 4B; *C380-Gal4* crossed to the genetic background control, *w^1118^*). Following spaced high K^+^ depolarization, we see 4-6 ghost boutons representing an approximately 3-fold increase (Figure 4B; p<0.0001) [14], [15]. As predicted, presynaptic expression of pri-miRNAs encoding for three of five activity-regulated miRNAs suppressed synaptic growth stimulated by spaced high K^+^ depolarization (in miR-8, -289, and -958 there was no significant increase in ghost bouton numbers compared to pseudostimulated controls). In contrast, two activity-regulated miRNAs appear to not play a role in ghost bouton formation at the NMJ. First, misexpression of miR-314 had no negative effect on synaptic growth (Figure 4B; p<0.05). Second, while miR-1 results trend towards a reduction in ghost bouton numbers following spaced stimulation (down to ∼ 3 per NMJ) the baseline was also reduced in pseudostimulated controls (Figure 4B; down to ∼1 per NMJ). Thus, as seen in control larvae, an activity-dependent 3-fold increase in ghost boutons was observed.

We next sought to confirm our miRNA misexpression results using available mutant and transgenic reduction-in-function lines. miR-1 mutants are embryonic or early larval lethal [36], [37]. In contrast, miR-8 mutants survive until the pupal stage and exhibit a robust phenotype at the third instar larval NMJ [19], [20]. Also available were transgenic miR-8 “sponge” constructs that contained 10 repetitive sequences of a strong miR-8 binding site under control of UAS elements (*UAS-miR-8SP*) [20]. We predicted that, if miR-8 is required for activity-dependent synaptic growth at the NMJ, then we would see one (or perhaps both) of two phenotypes: 1) a significant increase in the number of ghost boutons per NMJ in response to spaced stimulation compared to pseudostimulated controls; or 2) an enhancement in the ability of the NMJ to respond to spaced stimulation so that there would be a significant increase in ghost boutons per NMJ in response to fewer cycles of spaced stimulation (compared to the normal 5 cycles observed in controls; Figure 4B). As expected, we observed the latter when the expression of only one copy of the miR-8 sponge was driven in larval motor neurons (Figure 4B; *C380-Gal4/+;UAS-miR-8SP/+*; p<0.01). Although the result was not statistically significant, we observed a similar trend in miR-8 mutants (Figure 4B; *miR-8^Δ1^*/*miR-8^Δ2^*). miR-8 has also been shown to have a strong positive postsynaptic function in the control of total bouton number during NMJ development (Figure 4C; Figure S2C) [20]. Bearing this in mind, the global disruption of miR-8 may have a significant negative effect on activity-dependent synaptic growth.

The suppression of activity-dependent ghost bouton formation could be due to a general negative effect on synaptic growth during larval NMJ development. To examine this possibility, we assessed synapse size by counting the combined total number of synaptic boutons at the same NMJs assayed in Figure 4B. With one exception, we observed no effect on synaptic bouton numbers in any miRNA-misexpressing background compared to controls (Figure 4C; Figure S2A–C). Surprisingly, total bouton counts in miR-8 misexpressing lines were slightly but significantly increased (19% increase; p<0.001). This result was not expected given the reduction in ghost boutons observed following high K^+^ stimulation (Figure 4B). However, in support, miR-8 mutants exhibited a significant decrease in total bouton numbers compared to controls (Figure 4C; Figure S2C; *miR-8^Δ1^*/*miR-8^Δ2^*; 38% decrease; p<0.0001) [20]. We observed a very similar effect when one copy of the miR-8 sponge was driven in larval motor neurons (Figure 4C; Figure S2C; *UAS-miR-8SP x C380-Gal4*; 31% decrease; p<0.0001). Together, these data suggest that miRs-8, -289, and/or -958 may have specific functions in the control of activity-dependent synaptic growth. These observations also raise the interesting possibility that the mechanisms controlling activity-dependent processes can be uncoupled from those that control NMJ development (Figure 4B–C). In contrast, miRs-1 and -314 may also have specific activity-dependent functions in larval neurons, but not at this particular synapse.

### Gene Ontology (GO) Cluster Analysis of Target mRNAs

**Figure 5:**
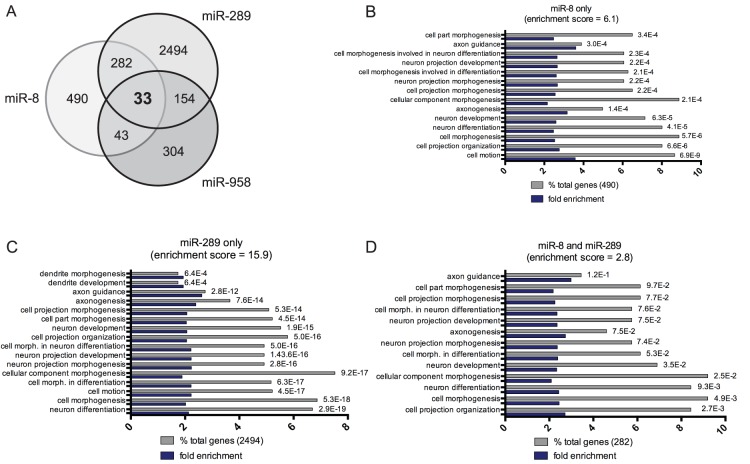
Predicted targets of miRs-8 and -289 are found in neuron-related enriched functional annotation clusters. (A) Venn diagram showing predicted mRNA targets for activity-regulated miRNAs. Notably, 33 mRNAs have putative binding sites for miRs-8, -289, and -958. 282 mRNAs have putative binding sites for miRs-8 and -289. (B-C) Functional annotation cluster analysis for predicted targets of miRs-8, -289 and both miRs-8 and -289. Only clusters enriched with targets significantly enriched in clusters involved in the control of neuronal processes are shown here. Note that the 304 predicted targets for miR-958 were not enriched in these clusters. Enrichment scores for each cluster are indicated [29], [30]. Genes within each category are indicated as a percentage of total genes (gray bar) and fold enrichment (blue bar) over expected number of genes in that category in the *Drosophila* genome. Statistical significance (Benjamini corrected p-values) for each category is indicated to right of gray columns.

One important role of miRNAs in neurons is believed to be the coordination or fine-tuning of mRNA expression pathways associated with neural plasticity [38], [39]. Bearing this in mind, we next combined *in silico* target mRNA identification with functional annotation cluster analysis to identify neuronal roles for miRs-8, -289, and/or -958 in the larval CNS [29], [30]. First, we used miRecords to identify putative mRNA target for each activity-regulated miRNA [28]. miRecords is advantageous because it cross-references results from 11 target prediction algorithms. This approach is based on the relatively simple hypothesis that valid miRNA/mRNA interactions are much more likely to be predicted by multiple databases. Using miRecords, we identified 490 putative mRNA targets for miR-8, 2494 for miR-289, and 304 for miR-958 (Figure 5A; Table S3). To reduce the size of each list, we then speculated that key mRNA targets encoding for proteins involved in the control of activity-dependent synaptic growth are likely to be co-regulated by miRs-8, -289, and/or -958. Therefore, we asked if there was any overlap between mRNA targets found in each group. We found that: a) 282 mRNAs are predicted targets of miRs-8 and -289; b) 43 of miRs-8 and -958, and c) 154 of miRs-289 and -958 (Table S4). Furthermore, we found that 33 mRNAs are predicted targets for co-regulation by miRs-8, -289, and -958 (Table S5). Second, we focused on identifying functionally related pools of target mRNAs within annotation clusters relating to neuron development, morphogenesis, and/or differentiation. Interestingly, we found that predicted targets for miRs-8 and -289 but not miR-958 were found in these enriched annotation clusters (Figure 5B–C; Table S3; enrichment scores = 6.1 and 15.9 respectively). Because these results suggest that both miRs-8 and -289 may be involved in the coordination of neuronal gene expression, we analyzed the 282 predicted co-regulated mRNA targets of miR-8 and miR-289 (Figure 5A; Table S4). Again, we found a statistically significant number that mapped to neuron-related clusters (Figure 5D; 32 mRNA targets; enrichment score = 2.8). In contrast, the 33 predicted mRNA targets for co-regulation by all three activity-regulated miRNAs did not show any significant neuronal enrichment (data not shown; Table S5). This is possibly due to the relatively high number of unannotated genes in this group. Taken together, these data provide support for two conclusions. First, functional annotation cluster analysis indicates that miRs-8, -289, and -958 are each involved in the control of a diverse array of pleiotrophic cellular processes during *Drosophila* development (data not shown). Second, both miRs-8 and -289 are strong candidates for the coordination of mRNA expression pathways relating to the control of a number of neuronal processes including, but not limited to, axon development, pathfinding, and growth.

### Identification of Biologically Relevant Target mRNAs

We next sought to identify target mRNAs encoding for proteins with potentially novel functions in the control of miRNA-mediated activity-dependent synaptic growth. We initially focused on genes with annotated functions in the control of axon physiology and/or neurite outgrowth that were found in one of two groups: 1) mRNAs targeted for co-regulation by miRs-8 and -289 that map to a neuron-related annotation cluster (Table 1); or 2) all mRNAs targeted for co-regulation by miRs-8, -289, and -958 (Table 2).

**Table 1:** Putative mRNA targets for co-regulation by miRs-8 and -289 that also map to a neuron-related functional annotation cluster (GO term enrichment).

Gene	Gene Name	Molecular Function
1ABI	Abelson Interacting Protein	protein binding
AP	apterous	DNA binding
B-H2	BarH2	transcription factor
2BAZ	bazooka	phosphatidylinositol binding
CBL	Dmel_CG7037	protein binding
CK	crinkled	actin-dependent ATPase activity
CRB	crumbs	protein kinase C binding
CSW	corkscrew	protein tyrosine phosphatase activity
DG	Dystroglycan	protein binding
DS	dachsous	cell adhesion molecule binding
FRY	furry	protein binding
GOGO	golden goal	receptor activity
HTH	homothorax	transcription factor activity
JUMU	jumeau	DNA binding
KAP3	Kinesin associated protein 3	protein binding
3LAR	Leukocyte-antigen-related-like	protein tyrosine phosphatase
4LOLA	longitudinals lacking	transcription factor
MID	midline	transcription factor
MLE	maleless	chromatin binding
PATJ	Patj homolog	protein kinase c binding
5PLEXA	plexin A	14-3-3 protein binding; semaphorin receptor
6PTP99A	Protein tyrosine phosphatase 99A	protein tyrosine phosphatase
PVF1	PDGF- and VEGF-related factor 1	heparin binding; receptor binding
7ROBO	roundabout	heparin binding; protein binding
RST	roughest	PDZ domain binding
8SEMA-1A	Semaphorin-1A	protein binding
SINA	seven in absentia	protein binding
SNA	snail	transcriptional repressor activity
VN	vein	heparin binding; receptor binding
9WG	wingless	frizzled-2 binding
10WIT	wishful thinking	TGFβ receptor
YKI	yorkie	protein binding; transcriptional coactivator activity

1 Presynaptic Ableson Interacting Protein (Abi) acts to antagonize the Abl tyrosine kinase (Abl) in the control of synaptogenesis and neurite extension at the larval NMJ [52].

2 A bazooka (Baz)/Par-6/aPKC complex controls new synaptic bouton growth at the NMJ [60].

3 The leukocyte-antigen-related like (Lar) controls synapse morphogenesis at the larval NMJ [16].

4 The longitudinals lacking (Lola) protein is required for embryonic axon growth and guidance [58].

5 Plexin A (PlexA) is a conserved neuronal semaphorin receptor that controls axon guidance [53].

6 The receptor tyrosine phosphatase (PTP99A) is required for embryonic motor axon guidance [46].

7 Roundabout (Robo) controls axon crossing of the embryonic CNS midline [45].

8 Semaphorin-1A (Sema-1A) is a ligand for PlexA and required for embryonic motor axon guidance [54].

9 Secreted wingless (Wg) protein is required for activity-dependent synapse growth at the NMJ [12].

10 The BMP type II receptor, wishful thinking (Wit), is required for axon terminal growth and synapse function at the larval NMJ [43], [44].

**Table 2:** Putative mRNA targets for co-regulation by miRs-8, -289, and -958 (miRBase).

Gene	Gene Name	Molecular Function
B4	Dmel_CG9239	unknown
CALPA	Calpain-A	calcium-dependent cysteine-type endopeptidase
1CG10077	Dmel_CG10077	RNA helicase activity
CG10731	Dmel_CG10731	hydrogen-exporting ATPase
CG1273	Dmel_CG1273	unknown
CG2519	Dmel_CG2519	unknown
CG31530	Dmel_CG31530	organic cation transmembrane transporter
CG32017	Dmel_CG32017	unknown
CG32365	Dmel_CG32365	unknown
CG32683	Dmel_CG32683	unknown
CG33981	Dmel_CG33981	unknown
CG4467	Dmel_CG4467	aminopeptidase
CG8475	Dmel_CCG8475	phosphorylase kinase regulator
CLK	Clock	transcription factor
CSW	corkscrew	protein tyrosine phosphatase
GUG	Grunge	histone deacetylase
H	Hairy	transcription corepressor activity
HTH	homothorax	transcription factor activity
2LAR	Leukocyte-antigen-related-like	protein tyrosine phosphatase
2LOLA	longitudinals lacking	transcription factor
LOOPIN-1	Loopin-1	aminopeptidase
PATJ	Patj homolog	protein kinase C binding
PLC21C	Phospholipase C at 21C	phosphatidylinositol phospholipase C
RBP9	RNA-binding protein 9	mRNA binding
RHOGEF3	Dmel_CG42378	Rho guanyl-nucleotide exchange factor
STAR1	allatostatin C receptor 1	allatostatin receptor
TOLLO	Tollo	transmembrane signaling receptor
UNC-4	Homeobox protein unc-4	transcription factor
UNK	unkempt	zinc ion binding; DNA binding
VACHT	Vesicular acetylcholine transporter	acetylcholine transmembrane transporter
VMAT	Vesicular monoamine transporter	synaptic vesicle amine transmembrane transporter
WRY	Weary	Notch binding
ZN72D	Zinc-finger protein at 72D	mRNA binding

1 CG10077 is the *Drosophila* ortholog of the mammalian DEAD box RNA helicase, DDX5, which has been identified as a component of kinesin-containing neuronal RNPs in mice [61].

2 Lar and Lola do have known functions in axonogenesis and are described in both the text and in Table 1.

Of the mRNAs we predicted to be co-regulated by miRs-8 and -289, 32 of 282 (11%) also map to a neuron-related enriched functional annotation cluster (Figure 5D; Table 1; Table S4). Of these, 10 (31%) have an annotated function in the control of axon development, guidance, and/or growth (Table 1). First, both the Wg and Wishful Thinking (Wit) proteins are components of highly conserved signaling pathways involved in the control of synaptic growth at the larval *Drosophila* NMJ [40]. Evoked synaptic activity induces Wg secretion from axon terminals where it can bind to both pre- and postsynaptic Frizzled-2 (Fz2) receptor and control activity-dependent modifications in synapse structure and function [12], [41]. Importantly, mutations that reduce Wg protein levels can completely prevent activity-dependent increases in ghost bouton formation following spaced high K^+^ depolarization [12]. In contrast, Wit is a presynaptic type-II BMP receptor that binds to the retrograde BMP ligand, Glass bottom boat (Gbb) [42]. While no role for Wit in activity-dependent synaptic growth has been characterized, larvae with mutations in *wit* do have abnormally small NMJs [43], [44]. Second, this group includes six mRNAs encoding for conserved components of axon guidance pathways. The receptor protein, Roundabout (Robo), is expressed on axon terminals, binds to the chemorepellent Slit, and controls axon crossing of the embryonic CNS midline [45]. The *Drosophila* receptor-linked protein tyrosine phosphatases (RPTPs), Lar and Protein tyrosine phosphatase 99A (PTP99A) are also expressed on axon terminals and are involved in pathfinding by motor neurons [46], [47], [48], [49]. Evidence suggests that Lar interacts negatively with the Ableson (Abl) tyrosine kinase to control motor neuron axon guidance and *lar* loss-of-function leads to a significant decrease in total bouton numbers at the larval NMJ [50], [51]. Interestingly, the Abl Interacting Protein (Abi) is also found in this group. Abi interacts with Enabled (Ena) to antagonize Abl function in synaptogenesis and neurite outgrowth [52]. Finally, Plexin A (PlexA) is a neuronal receptor for secreted class I semephorins including Semaphorin-1A (Sema-1A) and controls motor and CNS axon guidance [53], [54]. Presynaptic Sema-1A is required to form the embryonic NMJ, suggesting that Sema-1A can also act as a receptor on axon terminals [55]. A number of studies have implicated semaphorin family members as putative targets for regulation by the miRNA pathway [56]. Third, the Longitudinals Lacking (Lola) protein is a transcription factor involved in the control of embryonic CNS midline crossing by coordinating the regulation of both *slit* and *robo* expression [57], [58]. Lola also controls axon growth and guidance by suppressing expression of the actin nucleation factor, Spire (Spir) [59]. Finally, a complex containing Bazooka (Baz), Par-6, and atypical Protein Kinase C (aPKC) is required to control new synaptic bouton growth by regulating microtubule cytoskeleton dynamics [60]. Decreasing Baz levels decreases the number of synaptic boutons at the larval NMJ.

Of the 33 mRNAs co-regulated by miRs-8, -289, and -958, three (9%) have predicted or experimentally proven functions in the control of either axon or dendrite development (Table 2). First, as indicated above, both the *Drosophila* Lar and Lola proteins have characterized functions in the control of axon guidance and/or growth [16], [58]. Second, CG10077 is the fly ortholog of DDX5, a conserved DEAD-box RNA helicase that has been found to be a component of kinesin-containing transport RNPs in mouse neurons [61]. Beyond this, specific functions for DDX5 in neuronal RNPs have not yet been characterized. Interestingly, 11 (33%) of these putative co-regulated mRNAs are currently uncharacterized (indicated by a CG gene symbol; this number includes CG10077). These mRNAs represent a pool of potentially novel targets for miRNA-mediated regulation of activity-dependent synaptic growth at the larval NMJ.

### Experimental Validation of Putative Target miRNAs

The identification of miRNA targets using bioinformatic approaches does not indicate a genuine miRNA/mRNA interaction. Therefore, each putative target for co-regulation by activity-regulated miRNAs requires direct experimental validation. As proof of concept, we have focused here on two predicted target mRNAs of activity-regulated miRNAs: a) *lar* because it is required for axon guidance and/or synaptic growth and is a putative target for co-regulation by miRs-8, -289, and -958 (Tables 1 and 2) [16], [49]; and b) *wg* because it has previously been shown to be involved in the control of activity-dependent ghost bouton formation at the larval *Drosophila* NMJ (Table 1) [12].

**Figure 6:**
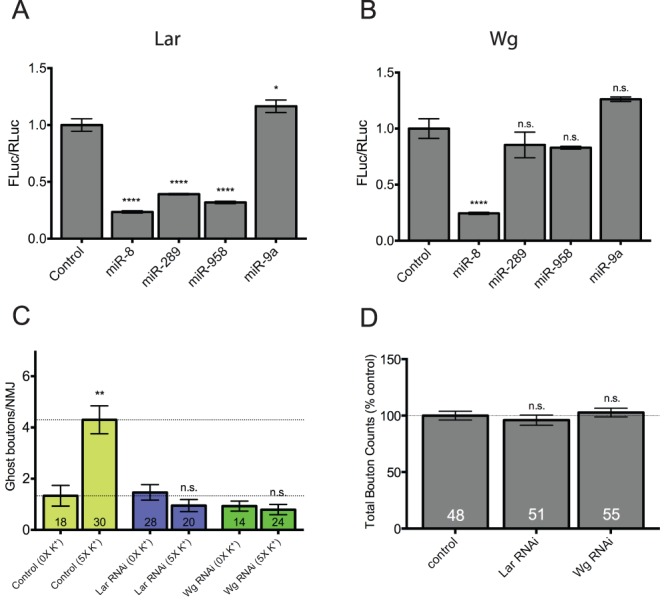
*Drosophila lar* and *wg* are authentic targets for translational repression by activity-regulated miRNAs. (A and B) Reporter plasmids constitutively expressing firefly luciferase (Fluc) flanked by the 3′UTRs of *lar* and *wg* were cotransfected into S2 cells with plasmids expressing the indicated miRNA primary transcripts as indicated. *Renilla* luciferase (Rluc) was included as a transfection control. Fluc activity was normalized to Rluc activity in three independent experiments. Normalized Fluc activities in the absence of miRNA-expressing vectors (emply vector controls) were set to 1. (A) The *Drosophila lar* 3′UTR has one binding site each for miRs-1, -8, -289, and -958. miR-1 was not tested because it did not negatively regulate activity-dependent synaptic growth. miRs-8, -289, and -958 all significantly repress *lar* Fluc reporter activity. In contrast, a miRNA with no predicted binding site (miR-9a) has no effect on Fluc activity. (B) The *wg* 3′UTR has three predicted binding sites for miR-289 and two for miR-8. Interestingly, only miR-8 is capable of repressing *wg* Fluc reporter activity. miR-289, -958 nor -9a (not predicted to bind) had an effect of Fluc activity. In both (A) and (B) error bars indicate the mean ± SEM (n = 3). (C) Transgenic hairpin RNAi constructs targeting *lar* and *wg* were misexpressed in larval motor neurons using the *C380-Gal4* driver (e.g. genotype *C380-Gal4/+; UAS-RNAi^hairpin^/+*). Note that both hairpin constructs completely prevented activity-dependent ghost bouton formation. (D) Quantification of the total number of synaptic boutons at the same NMJs assayed in above (C). Neither hairpin construct had a significant effect on total bouton numbers compared to controls (*C380-Gal4/+*) suggesting that activity-dependent processes may be more sensitive to disruption of genes involved in synaptic growth pathways. STATISTICS: (A-B; D) One-way ANOVA with a Dunnett’s post-hoc test. (C) Kruskal-Wallis multiple comparison analysis with a Dunn’s post-hoc test. n.s. = not significant ** p<0.01 **** p<0.0001.

*Lar* has one predicted binding site (each) for miRs-8, -289, and -958 in its 3′UTR (binding sites for miR-289 =  m8; miRs-8 and -958 = 7mer-m8). *Lar* also has one binding site for a fourth activity-regulated miRNA, miR-1, as well as members of the miR-310-313 cluster (Figure 3A-C; binding sites for miR-1 = 7mer-m8; miR-310–313 = 7mer–m8) [62]. In contrast, the 3′UTR of *wg* has three predicted binding sites for miR-289 and two for miR-8 (binding sites for miR-289 =  m8, m8, and 7mer-m8; mir-8 =  m8 and 7mer-1A). To determine if *lar* and *wg* represented genuine targets of activity-regulated miRNAs, we first cloned the *lar* and *wg* 3′UTRs into a firefly luciferase reporter vector [31]. When the *lar* reporter was co-transfected with each activity-regulated miRNA, we found that all three significantly repressed luciferase expression (Figure 6A; miR-8 = 24%; miR-289 = 39%; miR-958 = 32%; all at p<0.0001). In contrast, co-transfection with a miRNA not predicted to bind to the *lar* 3′UTR (miR-9a) had no effect on reporter expression. When the *wg* reporter was co-transfected with miRs-8, -289, or -958 we found that only miR-8 was capable of repression (Figure 6B; miR-8 = 24%; p<0.0001). Surprisingly, despite the presence of three predicted miR-289 binding sites (two of which exhibit a perfect seed pairing), miR-289 had no effect on reporter expression (Figure 6B). Again, as a negative control, co-transfection with a miRNA not predicted to bind to the *wg* 3′UTR (miR-9a) had no effect on luciferase reporter activity. Together, these data provide experimental support for *lar* and *wg* as potential mRNA targets for activity-regulated miRNAs and suggest that miRs-8, -289, and -958 may be controlling activity-dependent growth, in part, by regulating expression of Lar and/or Wg in motor neurons.

Unfortunately, good antibodies that recognized Lar and Wg in NMJ axon terminals were not readily available. However, to support our observations, we predicted that transgenic knockdown of *lar* and/or *wg* by RNAi would phenocopy miR-8, -289, and -958 misexpression and prevent activity-dependent synaptic growth. As previously shown, disruption of Wg expression completely prevented ghost bouton formation in response to spaced high K^+^ depolarization (Figure 6C; *UAS-wg^HMS00794^* x *C380-Gal4*) [12]. Disruption of Lar expression by RNAi also prevented activity-dependent axon terminal growth (Figure 6C; *UAS-lar^HMS00822^ x C380-Gal4*). Surprisingly, knockdown of neither *lar* or *wg* by RNAi was sufficient to affect synapse size during development of the larval NMJ (Figure 6D; Figure S3). Together, these data provide further support for the hypothesis that the regulation of synaptic growth during development can be mechanistically uncoupled from that following acute spaced synaptic stimulation. The former process appears to be somewhat less sensitive to the modulation of *lar*, *wg,* and miRNA (miR-289 and -958) levels than the latter.

## Discussion

The major conclusion of this study is that acute spaced synaptic stimulation controls the expression of specific miRNAs in the larval *Drosophila* CNS. In turn, several of these activity-regulated miRNAs are involved in the control of activity-dependent axon terminal growth at the NMJ. This conclusion is based on the following experimental evidence. First, we demonstrate that, in a screen of 62 neuronally expressed miRNAs, five (miRs-1, -8, -289, -314, and -958) exhibited a greater than 2-fold or statistically significant down-regulation following spaced high K+ depolarization (Figure 3A–C). Next, we show that neuronal misexpression of three activity-regulated miRNAs (miRs-8, -289, and -958) is sufficient to inhibit activity-dependent synaptic growth at the larval NMJ (Figure 4B). These observations are supported in miR-8 mutants and by miR-8 reduction-in-function (Figure 4B). Using bioinformatics to identify putative target mRNAs combined with functional annotation cluster analysis, we find that targets of miRs-8 and -289 (but not miR-958) are enriched in neuron-related clusters (Figure 5B–D). Interestingly, both miR-8 and -289 are predicted to target the mRNA encoding for the Wg protein (Table 1). Consistent with this observation, the downregulation of Wg expression levels by RNAi and in *wg* mutants has been shown to inhibit activity-dependent ghost bouton formation in response to spaced high K+ stimulation (Figure 4A; 6C) [12]. Finally, we provide evidence indicating that miRs-289 and -958 can regulate the expression a *lar* 3′UTR reporter, and miR-8 can regulate the expression of both a *lar* and *wg* 3′UTR reporter *in vitro* (Figure 6A–B). Taken together, these data suggest that miRs-8, -289, and possibly -958 may be involved in coordinating the expression of genes involved in activity-dependent synaptic growth at the NMJ.

### miRNA-mediated Control of Synaptic Plasticity in *Drosophila*

Three lines of evidence support the hypothesis that miRNAs play important roles in the presynaptic control of synapse structure and/or function at the larval *Drosophila* NMJ. First, the miRNA effector protein, Argonaute 1 (Ago1), has previously been shown to interact with the fragile X mental retardation protein (FMRP) to regulate NMJ growth during larval development [63]. Second, the miR-310–313 cluster has been found to control normal synaptic transmission at the *Drosophila* NMJ via presynaptic regulation of the Kinesin family member, Kinesin-73 (Khc-73) [62]. Third, miR-124 loss of function increases synaptic release at the NMJ, presumably by coordinating the repression of mRNAs encoding for components in the BMP signaling pathway including the BMP receptors Wit and Saxaphone (Sax), and the downstream transcription factor Mothers against dpp (Mad) [64]. In addition, two studies show that miRNAs have postsynaptic functions in the control of synaptic plasticity. First, postsynaptic downregulation of miR-8 levels (using a miR-8 sponge) results in a significant decrease in synapse size during development of the larval NMJ [20]. Second, postsynaptic miR-284 is required to regulate glutamate receptor (GluR) availability at the NMJ [65]. In support, postsynaptic RNAi-mediated knockdown of Dicer-1 (Dcr-1), the fly endonuclease responsible for miRNA biosynthesis, leads to the upregulation of postsynaptic GluR mRNA and protein levels. Taken together, these data provide strong evidence indicating a function for the miRNA pathway in coordinating the expression of genes involved in the pre- and postsynaptic control of plasticity during NMJ development. However, despite this progress, little is known about how miRNAs regulate the expression of mRNAs involved in activity-dependent synaptic growth. The present study is the first to demonstrate that specific miRNAs have an activity-dependent function in the control of these processes.

### Activity-dependent Control of Synaptic Plasticity in Flies and Mammals

Two activity-regulated miRNAs identified in our screen (miRs-8 and -289) have previously been shown to have roles in the control of synapse structure and/or function. First, the miRNA pathway was found to be involved in the control of the activity-dependent translation of postsynaptic *calcium/calmodulin-dependent protein kinase II* (*CamKII*) mRNA in the adult *Drosophila* antennal lobe [66]. Although no direct involvement was demonstrated, the *CamKII* 3′UTR does have one predicted binding site for miR-289. Second, as indicated above, miR-8 has been shown to have a postsynaptic function in the control of synapse size during development of the larval NMJ [20]. Although presynaptic functions for miRs-8 and -289 have not yet been identified, activity-dependent synaptic growth has not been specifically assayed.

Activity-regulated miRNAs appear to fall into two general categories (Figure 3C). miRs-958 and -314 show a very strong decrease in mature miRNA levels following acute spaced stimulation (7.5- and 5.2-fold respectively). In contrast, miRs-289, -1, and -8 show a somewhat lesser effect (2.5-, 2.4-, and 1.8-fold decrease). Two miRNAs that fall into the latter group (miRs-1 and -8) belong to gene families conserved from flies to humans [67]. In contrast, miRs-289, -314, and -958 appear to be members of families unique to species of Drosophilids. This final observation does raise questions about the applicability of miR-289 and -958 results to analogous studies in higher organisms.

Generally speaking, work in mammals has shown that synaptic activity leads to the rapid upregulation of key miRNAs that, in turn, negatively regulate the translation of mRNAs involved in the control of synapse structure and/or function [39]. Why did we not identify any significantly upregulated miRNAs in our experiments? First, we have screened only ∼35% off all currently annotated *Drosophila* miRNAs (Figure 2A–B). The most likely explanation is that we missed one or more miRNA that is both upregulated by activity and required for activity-dependent synaptic growth. Second, of the mammalian miRNAs identified thus far (miRs-29a/b, -132, -134, and -188) only miR-29a/b has a *Drosophila* family member (miR-285). Fly miR-285 has no known function and was not identified as a miRNA expressed in the larval CNS (Table S1). Third, downregulation of miRNA levels could be an indirect consequence of the high K^+^ stimulation paradigm. However, we do feel this is unlikely because the results of our high K^+^ RT-qPCR screen significantly correlate with those from our ChR2 light-stimulated miRNA array (across examined miRNAs; Figure S1). Finally, this could represent a genuine difference between fly and mammalian neurons.

### Mechanisms of Activity-dependent miRNA Down-regulation

What mechanisms are involved in the activity-dependent decrease in mature miRNA levels? Two lines of evidence suggest that this is due to rapid transcriptional downregulation. First, miRs-12 and -304, miRNAs presumably co-regulated in a miRNA cluster, show identical levels of downregulation following spaced synaptic stimulation (Figure 3B–C) [34]. Second, misexpression of key transgenic pri-miRNAs (specifically miRs-8, -289, and -958) that cannot be regulated at the level of transcription can suppress activity-dependent synaptic growth (Figure 4B). One would expect that these transgenic miRNAs would be equally affected by a mechanism that negatively affects mature miRNA stability. Despite this, there is some evidence from the mouse optic lobe that synaptic activity (i.e. dark adaptation) can lead to the rapid downregulation of specific miRNAs via miRNA decay [68]. Alternatively, there is also evidence that processing of specific miRNAs can be inhibited *in vivo*. For example, the conserved RNA binding protein, Lin28, can specifically bind to the let-7 pre-miRNA and negatively regulate let-7 biogenesis [69]. It would be interesting to determine if either mechanism were involved in activity-dependent miRNA downregulation in the larval CNS.

### Conclusions

In summary, the data presented in this study suggest that specific miRNAs coordinate the expression of genes involved in the control of activity-dependent synaptic growth at the larval *Drosophila* NMJ. Interestingly, a significant number of predicted target mRNAs encode for guidance molecules in conserved axon pathfinding pathways. A growing number of studies have implicated axon guidance molecules as critical regulators of synapse formation and plasticity [70]. Further analysis is needed in order to identify and characterize: a) additional activity-regulated miRNAs; and b) *bona fide* mRNA targets. Importantly, these targets need to be validated both *in vitro* and *in vivo* and their role in miRNA-mediated activity-dependent axon terminal growth confirmed.

## Supporting Information

Figure S1 **Correlation between ChR2 light and high K^+^ stimulation paradigms.** Regression analysis between calculated fold expression levels of all miRNAs analyzed in both the ChR2 light-induced miRNA microarray and high K^+^-induced RT-qPCR experiments. The fitted regression line is shown. There is a highly statistically significant correlation in expression levels across all miRNAs analyzed (p<0.001).
(EPS)

Figure S2 **Some activity-regulated miRNAs also control synaptic growth during larval development.** (A-B) At the same NMJs analyzed in Figure 4C, type 1b (big) and 1s (small) boutons were quantified. Each type of bouton is derived from a distinct motor neuron [71]. Type 1b boutons are highly plastic and can be easily distinguished by their larger size and higher levels of the postsynaptic density marker, Dlg [72]. (A) miR-289 overexpression caused a significant increase in type 1b boutons compared to controls (17% increase; p<0.05). (A-B) miR-8 overexpression caused a significant increase in type 1s boutons compared to controls (29% increase; p<0.01). In contrast, miR-8 knockdown (*UAS-miR-8SP*) and deletion (*mir-8^Δ1^/^Δ2^*) larvae showed a significant decrease in both type 1b (31% and 38% decrease respectively; p<0.0001) and type 1s boutons (15% and 38% decrease respectively; p<0.0001). As seen with total boutons, high K^+^ stimulation has no effect on 1b or 1s bouton numbers compared to unstimulated controls (data not shown). The latter would have been indicative of spaced training affecting ghost bouton maturation or development. OE = overexpression; ROF = reduction-in-function; LOF = loss-of-function. Error bars indicate the mean ± SEM. (A-B) STATISTICS: Kruskal-Wallis multiple comparison analysis with a Dunn’s post-hoc test. * p<0.05 ** p<0.01 **** p<0.0001. (C) Representative third-instar NMJs from the indicated genotypes at larval muscle 6/7 in abdominal segment 3 that were stained with antibodies against the postsynaptic marker discs large (DLG). Note that the NMJs in both presynaptic miR-8 knockdown (*UAS-miR-8SP*) and deletion (*mir-8^Δ1^/^Δ2^*) larvae are phenotypically very similar and are significantly smaller than controls. In contrast, miR-8 overexpression NMJs exhibit some synaptic hyperplasia. Scale bars = 20 µM.
(EPS)

Figure S3 ***lar*** **and** ***wg*** **RNAi has no effect on synaptic growth during development.** (A-B) At the same NMJs analyzed in Figure 6D, type 1b (big) and 1s (small) boutons were quantified. Note that disruption of *lar* and *wg* expression using transgenic hairpin RNAi constructs had no specific effect on either type 1b or 1s boutons. The degree of Lar and Wg reduction was not confirmed. Error bars indicate the mean ± SEM. (A-B) STATISTICS: One-way ANOVA with a Dunnett’s post-hoc test. n.s. = not significant.
(EPS)

Table S1 **Summary of raw miRNA microarray data.** The relative change in expression levels on a miRBase 14.0 array for all detected miRNAs across treatment groups is indicated. N/A indicates a miRNA in the microarray that was not expressed at detectable levels. The full microarray data can be found in the Gene Expression Omnibus (GEO) of NCBI through accession number GSE43945.
(XLSX)

Table S2 **Summary of raw miRNA RT-qPCR data.** The relative change in expression levels for all miRNAs tested across treatment groups is indicated. BAD = primer pair that failed QC analysis. EXCLUDED = primer pair that failed to amplify in all biological replicates. The full RT-qPCR data can be found in the Gene Expression Omnibus (GEO) of NCBI through accession number GSE43945.
(XLSX)

Table S3 **Predicted targets of miR-8, -289, and -958.** A list of the 490, 2494, and 304 predicted mRNA targets for regulation by miRs-8, -289, and -958 (individually).
(XLSX)

Table S4 **Predicted targets of paired activity-regulated miRNAs.** A list of the 282 predicted mRNA targets for co-regulation by both miRs-8 and -289; 154 predicted targets of both miRs-289 and -958; and 43 predicted targets of both miRs-8 and -958.
(XLSX)

Table S5 **Predicted targets of all activity-regulated miRNAs.** A list of the 33 predicted mRNA targets for co-regulation by miRs-8, -289, and -958.
(XLSX)
